# Systematic Development of Self-Emulsifying Drug Delivery Systems of Atorvastatin with Improved Bioavailability Potential

**DOI:** 10.3797/scipharm.1201-06

**Published:** 2012-07-22

**Authors:** Fariba Khan, Md. Saiful Islam, Monzurul Amin Roni, Reza-Ul Jalil

**Affiliations:** 1 Department of Pharmacy, The University of Asia Pacific, Dhaka-1209, Bangladesh.; 2 Department of Pharmaceutical Technology, Faculty of Pharmacy, University of Dhaka, Dhaka-1000, Bangladesh.; 3 Department of Pharmacy, State University of Bangladesh, Dhaka-1205, Bangladesh.

**Keywords:** Self-emulsifying drug delivery systems, Atorvastatin, Oleic acid, Tween 80, Polyethyleneglycol 400

## Abstract

The aim of this study was to prepare and characterize a self-emulsifying drug delivery system (SEDDS) with a high drug load of poorly water-soluble atorvastatin for the enhancement of dissolution and oral bioavailability. Solubility of atorvastatin in oil, surfactant, and cosurfactant was determined. Pseudo-ternary phase diagrams were constructed by the aqueous titration method, and formulations were developed based on the optimum excipient combinations. A high drug load (10% w/w) was achieved with a combination of oleic acid, Tween 80, and polyethylene glycol 400, ensuring the maximum dissolution property (in the case of SES6). Effects of lipids and surfactants on physical properties of SEDDS such as *in vitro* emulsification efficiency in terms of self-emulsification time, emulsion droplet size, and percent transmittance were measured. Multiple regression analysis revealed that a higher amount of surfactants significantly increased dissolution of ATV while decreasing emulsion droplet size and emulsification time. About a four-fold increase in dissolution was achieved by SEDDS compared to pure ATV powder. Overall, this study suggests that dissolution and oral bioavailability of ATV could be improved by SEDDS technology.

## Introduction

Lipid-based formulation approaches, particularly the self-emulsifying drug delivery system (SEDDS), are well-known for their potential as alternative strategies for the delivery of hydrophobic drugs, which are associated with poor water solubility and low oral bioavailability [1–4]. SEDDS formulations are isotropic mixtures of an oil, a surfactant, a cosurfactant (or solubilizer), and a drug. The unique feature of this system is its ability to form fine oil-in-water (o/w) microemulsions under gentle agitation, following dilution by aqueous phases (e.g. the digestive motility of the stomach and intestine provide the agitation required for self-emulsification *in vivo* in the lumen of the gut) [5]. This spontaneous formation of an emulsion in the gastrointestinal tract presents the drug in a solubilized form, and the small size of the formed droplet provides a large interfacial surface area for drug absorption [6]. Apart from solubilization, the presence of lipid in the formulation further helps to improve bioavailability by affecting the drug absorption [1]. Selection of a suitable self-emulsifying formulation thus depends upon the assessment of the solubility of the drug in various components, the area of the self-emulsifying region as obtained in the phase diagram, and the droplet size distribution of the resultant emulsion following self-emulsification [7].

Atorvastatin (ATV), a 3-hydroxy-3-methylglutaryl coenzyme A (HMG-CoA) reductase inhibitor, is a plasma lipid-regulating agent. ATV has therapeutic application in hyperlipidemia and cardiovascular events. Oral bioavailability of ATV is only 12% and poor bioavailability has been attributed to its poor solubility in water and high presystemic clearance (> 80%) [8]. Recently, the utility of SEDDS in improving the dissolution and bioavailability of ATV has been reported [9–12]. However, the drug load in those studies was less than 5%. Higher drug load would lead to better patient compliance by reducing the size of the final dosage form. The aim of the present study was to prepare and characterize SEDDS formulations containing higher amounts of ATV and compare the dissolution properties with the pure powdered drug.

Response surface methodology (RSM) is used when only a few significant factors are involved. There are a good number of approaches available to achieve RSM, namely a three-level factorial design [13], central composite design (CCD) [14], Box-Bekhen design [15], and D-optimal design [16, 17], etc. But a reduced 3^2^ factorial design (without replicate) is also an established method to study the effect of selected parameters, and the rationale of using this method has been depicted previously [18, 19]. We used a reduced 3^2^ factorial design (without replicate) to characterize the optimized SEDDS, where the two independent variables and four dependent variables were considered.

## Experimental

### Materials

ATV calcium was purchased from Ranbaxy, India. Oleic acid (Merck, Germany), Arachis Oil (BDH Chemicals Ltd, Poole, England), Soybean oil (local brand), Castor oil (Merck, Germany), Glycerol (Shanghai Reagent Inc., China), Capmul PG 8 (Abitec Corporation, Germany), and Tween 20 and 80 (BDH Chemicals Ltd, England) were obtained as a gift. Cremophor CO 40, Cremophor CO 60, Cremophor EL, Cremophor RH 40, Cremophor RH 60, and polyethyleneglycol 400 (PEG 400) were received from BASF (Germany) as a gift.

### HPLC analysis

ATV was assayed by reverse-phase high-performance liquid chromatography (HPLC, Shimadzu Prominence LC 20 AT, Japan) equipped with the Pinnacle II C18 column (150mm× 4.5 mm, 5 μm). The mobile phase consisted of an ammonium acetate buffer (pH of 4.0)-acetonitrile solution (55:45) and pumped at a flow rate of 1.5 ml/min. The UV detector (Shimadzu Prominence SPD 20A UV/VIS detector, Japan) was set at 248 nm. The SEDDS formulations were dispersed in absolute methanol and centrifuged at 12000 rpm for 15 min. The supernatant sample (20 μL) was injected into the HPLC system.

### Solubility Screening

A solubility test of ATV was performed in various oils, surfactants, and cosurfactants. Briefly, 2 mL of oils, surfactants, or co-surfactants were taken in cap tubes containing excess ATV (500 mg). The samples were heated at ≤ 90°C for ≤ 5 min in a water bath and mixed by a vortex mixer. Resulting mixtures were then shaken in a thermal shaker (Memmert GmbH & Co. KG, Germany) at 50 rpm and 25°C for 48 h. Mixtures were equilibrated for 24 h at room temperature and then centrifuged at 3000 rpm for 10 min. The supernatant was collected and filtered using a membrane filter (0.45μm). The concentration of ATV was then quantified by the above-mentioned HPLC method.

### Pseudo-Ternary Phase Diagram

Pseudo-ternary phase diagrams of OA, S/CoS mixture, and water were developed using the water titration method. The mixtures of OA and S/CoS at certain weight ratios were diluted with distilled water in a drop-wise manner, where the S/CoS mixture was prepared using Tween 80 and PEG 400 at 1:1, 2:1, 3:1, and 5:1 ratios. For each phase diagram, a transparent and homogenous mixture of OA and S/CoS was formed by vortexing for 5 min. Then each mixture was titrated with water and visually observed for phase clarity and flowability. The amount of water at which turbidity-to-transparency and transparency-toturbidity transitions occurred was derived from the weight measurements. These values were then used to determine the boundaries of the microemulsion area corresponding to the values of OA and S/CoS. Phase diagrams were also constructed with ATV, where ATV-enriched OA was used as the oil phase. Phase diagrams were constructed using SigmaPlot 10.0 software (SPSS Inc., USA).

### Preparation of SEDDS

Optimum ratios of OA and S/CoS were selected from the phase diagrams. SEDDS formulations were prepared by dissolving ATV in S/CoS mixtures along with gentle vortexing and heating at ≤ 90 °C, and then by adding OA [20]. To study the effects of the formulation variables, different batches were prepared using 3^2^ factorial designs, with each batch containing 100 mg of ATV and varying amounts of OA and S/CoS (Table 1). Formulations were stored in a desiccator at ambient conditions for further study.

### In vitro Characterization of Optimized SEDDS

#### Assessment of Emulsification Time

The emulsification time of SEDDS formulations was determined in a USP dissolution tester (Electrolab, India). The SEDDS formulation equivalent to 10 mg of ATV was added drop-wise to 500 mL of distilled water maintained at 37 ± 0.5°C. Gentle agitation was provided by a paddle rotating at 50 rpm. The emulsification time was recorded manually [21].

#### Emulsion Droplet Size Determination

Emulsion droplet size was determined by the Malvern particle size analyzer (Mastersizer 2000, Malvern, UK). Briefly, SEDDS formulations (equivalent to 10 mg ATV) were diluted with 500 mL distilled water and thereafter, the droplet size was immediately determined. Each determination was done in triplicate.

#### Spectroscopic Characterization of Optical Clarity

Each formulation equivalent to 10 mg ATV was diluted with 500 mL of distilled water. The absorbance values of each emulsion at 0, 10, 20, and 30 min post-dilutions were measured by a UV spectrophotometer (UV mini-1240, Shimadzu, Japan) at 400 nm [22].

#### In Vitro Dissolution Studies

*In vitro* dissolution studies of ATV-SEDDS were conducted using the USP apparatus II (Electrolab, India) at a rotation speed of 50 rpm. For dissolution purposes, SEDDS formulations equivalent to 10 mg of ATV were filled in hard gelatin capsules (size # 3). Distilled water was used as the dissolution medium. The volume and temperature of the dissolution medium were 1000 mL and 37 ± 0.5 °C, respectively. Samples were withdrawn at fixed intervals, filtered with a 0.45 μm syringe filter (Microsart® Hannover, Germany), and analyzed for drug content by the HPLC method mentioned earlier.

#### Stability Study

Optimized SEDDS formulations were subjected to a stability study in a stability chamber (Thermolab, India) at accelerated conditions of 40°C/75% relative humidity, for over a period of three months. Samples were withdrawn at regular intervals and were considered for visual analysis.

#### RSM method

The data were subjected to multiple regression analysis and the response surface plots were constructed using Design Expert 8.0 (Stat-Ease Inc., USA). The data were fitted in equation 1,

Eq. 1 $$\text{Y}=\beta_{0}+\beta_{1}{\text{X}}_{1}+\beta_{2}{\text{X}}_{2}+\beta_{12}{\text{X}}_{1}{\text{X}}_{2}+\beta_{11}{\text{X}}_{11}+\beta_{22}{\text{X}}_{2}^{2}$$

where the independent variables (X) were the amounts of OA and S/CoS mixture, while the dependent variables (Y) were emulsion droplet size, percent transmittance, self-emulsification time, and percent ATV released after 10 min.

## Results and Discussions

### Solubility study

The aqueous solubility of ATV was very poor in water (0.02–1.21 mg/ml) and solubility was pH-dependent (Table 2). The solubility was improved in lipid vehicles, surfactants, and cosurfactants (Table 2). OA, Tween 80, and PEG 400 exhibited higher solubility than other vehicles. These three excipients were selected for further studies, where OA was chosen as the oil phase, Tween 80 as the surfactant, and PEG 400 as the cosurfactant. OA is an amphiphilic compound with surfactant properties, which is progressively and effectively replacing the regular medium chain triglyceride oils in SEDDS [1, 23, 24]. Tween 80 is one of the most widely recommended nonionic hydrophilic surfactants due to its relatively high hydrophilic-lipophilic balance value (HLB 15) and safety profile.

### Pseudo-ternary phase diagram study

Pseudo-ternary phase diagrams were constructed to determine the area of the micro-emulsion region. Self-microemulsifying systems form fine oil-water emulsions from only gentle agitation, upon their introduction into aqueous media. Surfactant and cosurfactant are preferentially adsorbed at the interface, reducing the interfacial energy as well as providing a mechanical barrier to coalescence. The decrease in the free energy required for the emulsion formation consequently improves the thermodynamic stability of the microemulsion formulation [20, 25].

Therefore, the selection of oil and surfactant, and the mixing ratio of oil to S/CoS, play an important role in the formation of the microemulsion. In the present study, four ratios of S/CoS were considered preliminary.

**Fig. 1.**
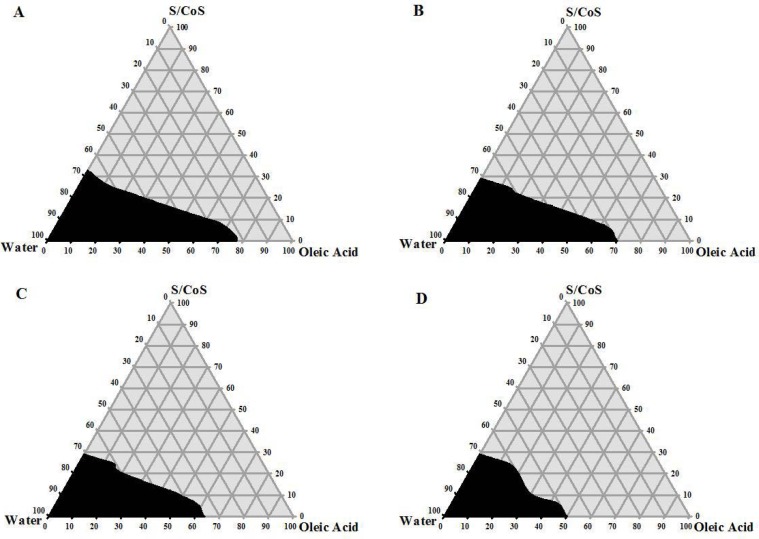
Pseudo-ternary phase diagrams of SEDDS where oil = oleic acid, surfactant = Tween 80, cosurfactant = PEG 400, and S/CoS means surfactant/cosurfactant mixture. S/CoS for A is 5:1, B is 3:1, C is 2:1, and D is 1:1.

**Fig. 2.**
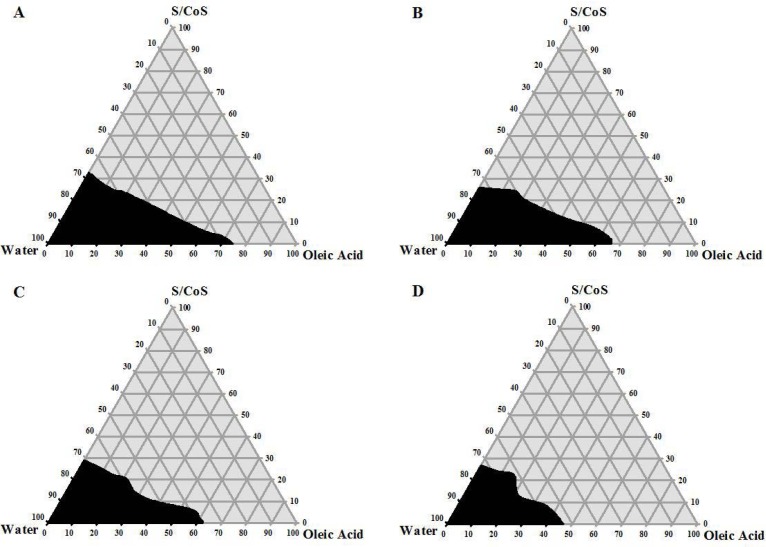
Pseudo-ternary phase diagrams of ATV-SEDDS where oil = atorvastatin-enriched oleic acid, surfactant = Tween 80, co-surfactant = PEG 400, and S/CoS means surfactant/co-surfactant mixture. S/CoS for A is 5:1, B is 3:1, C is 2:1, and D is 1:1.

Figure 1 shows the phase diagrams using four different ratios of S/CoS. Darker regions indicate the microemulsion area. A wider microemulsion area was observed with a S/CoS ratio of 5:1 (Figure 1A). As the S/CoS ratio was changed to 3:1, the microemulsion area became smaller and this narrowing of area was more distinguishable while the ratio was 2:1 and 1:1. A higher concentration of PEG 400 resulted in a narrow microemulsion area (Figure 1B, 1C, and 1D). This might be due to the hydrophilic nature of PEG 400, which caused a less efficient self-emulsification process. Though it helped more ATV to be dissolved, it failed to produce a wider microemulsion area [26]. The phase diagrams remained identical when constructed in the presence of ATV (Figure 2). Finally, the S/CoS mixture of the 5:1 ratio was selected for the formulation of SEDDS, as a larger microemulsion area indicates greater self-micro-emulsification efficiency, and a 5:1 ratio of S/CoS formed the largest microemulsion area. Another reason for selecting this combination of S/CoS was the presence of a lower amount of PEG 400. Mexi *et al.* reported that PEG 400 is incompatible with hard gelatin capsules when used in high concentrations, and there is always a chance of instability in SEDDS formulations which are intended to be filled in hard gelatin capsule shells [21].

Thereafter, using the optimum concentrations of the excipients, ATV SEDDS were prepared using the method mentioned earlier. During preparation, initially a temperature of ≤ 90°C was used for ≤ 5 min to avoid the chance of initial lump formation, which is a very common problem particularly when a high drug load (in this experiment, maximum 16.67% in case SES4) is used. According to a US patent report, atorvastatin salt remains stable above 60°C without any degradation [27]. Reports have also been published about the fact that atorvastatin calcium salt can retain its integrity, which is nearly similar to the innovator product’s behavior even after heating at 80°C for two days or 121°C (autoclaving) for 15 minutes [28, 29]. Rajasekaran & co-workers reported about only 7% degradation of atorvastatin calcium salt in the dry state while heating at 120°C for two hours [30]. In addition, another piece of evidence supporting the stability of ATV molecule within the formulated batches, can be its retention time (RT) [29]. In the case of pure ATV and the optimized batches, nearly the same RT was found.

### Characteristics of Optimized SEDDS

The performance of SEDDS was evaluated with respect to emulsification time, microemulsion droplet size, percent transmittance, and *in vitro* dissolution of ATV (Table 1).

### Emulsification Time

**Fig. 3.**
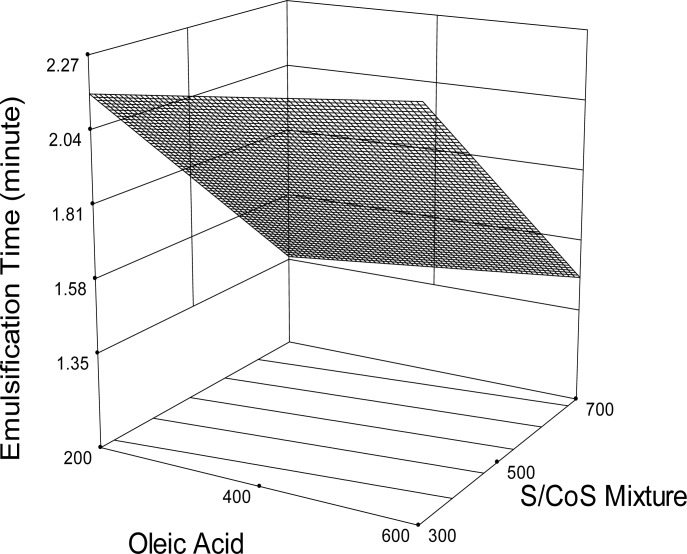
Response surface plot (3D) for the effects of oleic acid and surfactant/cosurfactant mixture (S/CoS) on emulsification time.

Emulsification time is an important index for the assessment of the efficiency of emulsion formation. SEDDS should disperse completely and rapidly when subjected to aqueous dilution under mild agitation. Emulsification time of the optimized SEDDS formulations is shown in Table 1. The formulation containing a higher amount of S/CoS took less time to be emulsified. Emulsification time decreased from 2.23 min to 1.32 min while the S/CoS concentration was increased from 300 mg to 700 mg. It might be due to the presence of a higher concentration of surfactant, which facilitated the self-emulsification process that eventually led to a high emulsification rate [19; 31]. Regression analysis revealed that OA and the surfactant mixture had a significant effect on emulsification time (P <0.0001). The response surface plot showed that formulations containing a higher amount of S/CoS exhibited a lower emulsification time (Figure 3).

### Microemulsion Droplet Size

**Fig. 4.**
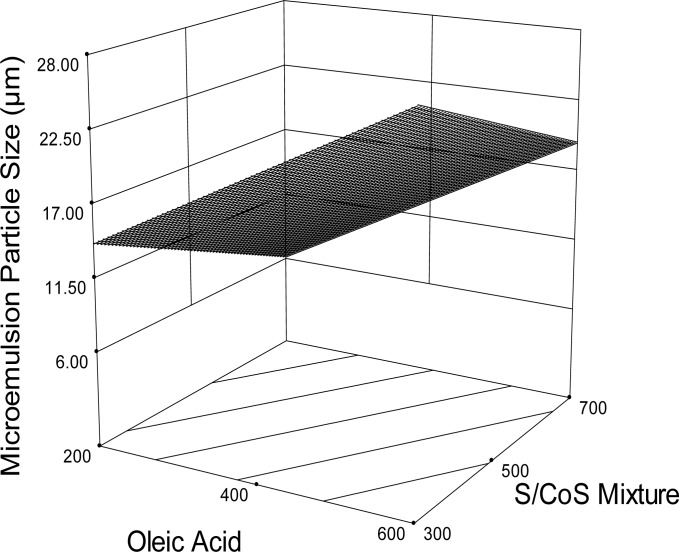
Response surface plot (3D) for the effects of oleic acid and surfactant/cosurfactant mixture (S/CoS) on microemulsion droplet size.

Microemulsion droplet size was within the range of 8.43 to 27.6 μm (Table 1), which is slightly larger than usual compared with available literature reports. The presence of ATV in a very large amount might result in larger emulsion droplets, and except in this experiment, such a high drug load was never attempted before. Reports have also been published about the fact that self-emulsification behavior of the oil phase may sometimes be altered if it contains drug [26]. Besides, relative concentrations of oil, surfactant, and cosurfactant also play a major role in microemulsion droplet size determination. For example, consider formulas SES6 & SES7 where the drug load is the same, 10%. But, SES6 produced smaller droplets than SES7. The presence of 70% of the S/CoS mixture in SES6 resulted in finer emulsion droplets. On the other hand, SES7 produced larger emulsion droplets, as it was comprised of only 30% of the S/CoS mixture. Gursory and Benita also reported that an increased amount of surfactant concentration led to droplets with smaller mean droplet size [31]. This may be explained by the fact that stabilization of the oil droplets is a result of the localization of the surfactant molecules at the oil-water interface [32]. Regression analysis and the response surface plot (Figure 4) revealed that droplet size was decreased with increasing surfactant concentration. The smallest droplet was found when 200 mg OA and 700 mg of S/CoS were used (SES6).

### Optical Clarity of the Optimized SEDDS

Optical clarity may be checked visually. But in order to measure it quantitatively, a UV-visible spectrophotometer was used to measure the amount of light of a given wavelength transmitted by the solution. Since cloudier solutions will scatter more of the incident light, resulting in lower transmittance values, higher transmittance should be obtained with optically clear solutions.

**Fig. 5.**
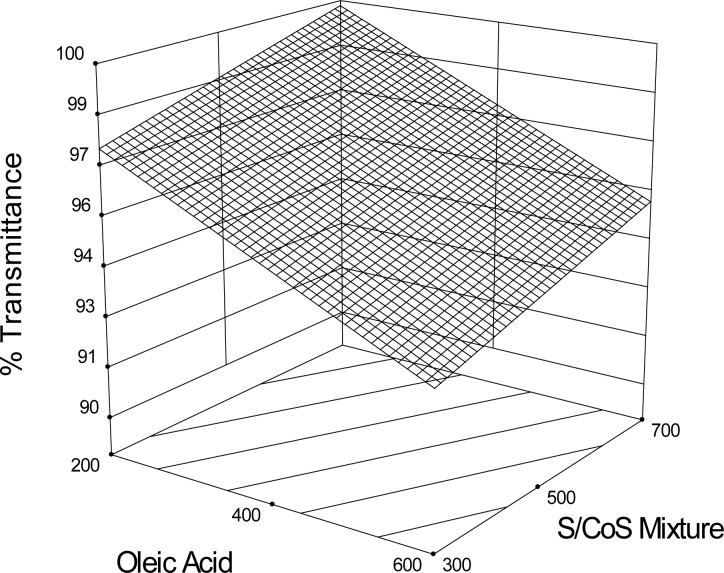
Response surface plot (3D) for the effects of oleic acid and surfactant/cosurfactant mixture (S/CoS) on optical clarity/transmittance capacity of the optimized batches.

All formulated batches were transparent (transmittance > 93%) (Table 1). The maximum transmittance value was found for SES6 (99.19%), indicating the formation of the microemulsion of the finest droplets. Figure 5 shows the 3D response surface plot of transmittance. Percent transmittance values of SEDDS were also measured at 10, 20, and 30 min after the dilution. The transmittance values remained unchanged even after 30 min of dilution (data not shown) which may be considered as a primary indication about the fact that the optimized SEDDS batches were stable.

### Release Profile of ATV

Atorvastatin shows pH-dependent solubility, and reports have been published previously where *in vitro* dissolution of atorvastatin calcium was studied in solutions of different pH, like simulated gastric fluid and simulated intestinal fluid, etc. [11, 33]. But we chose distilled water as the dissolution medium in this experiment. The reason was simply to investigate the solubility enhancing behavior of the optimized SEDDS batches of ATV in pure distilled water. In fact, to use distilled water for *in vitro* dissolution purposes of ATV is a significant move, as ATV shows very poor water solubility.

Talegaonkar *et al.* also reported about using distilled water as the dissolution medium and they successfully showed that solubility of ATV can be enhanced significantly by incorporating the drug in SEDDS [12].

**Fig. 6.**
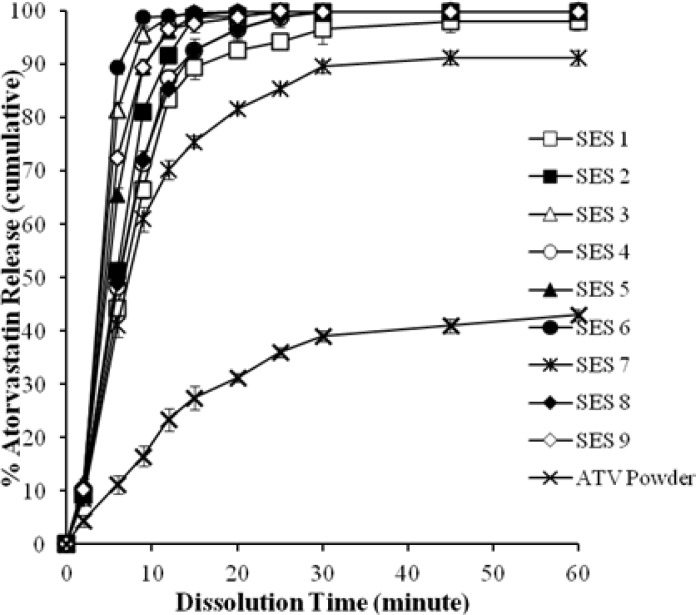
Comparative release profile of ATV from optimized batches of SEDDS and pure ATV powder.

**Fig. 7.**
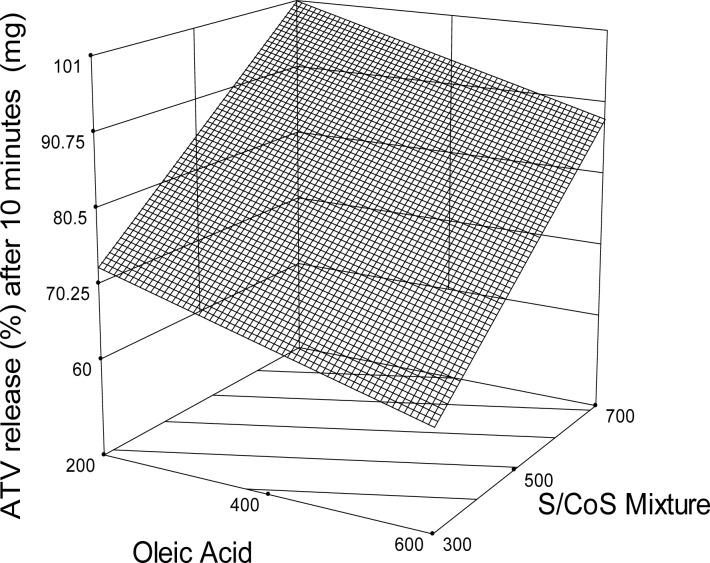
Response surface plot (3D) for the effects of oleic acid and surfactant/cosurfactant mixture (S/CoS) on atorvastatin-release (%) after 10 min.

Figure 6 shows the cumulative percent release of ATV from nine formulations and pure powdered ATV. The highest rate (about 90% in 5 min) of ATV dissolution was obtained from the formulation (SES6) containing 200 mg of OA and 700 mg of surfactant mixture. Regression analysis (Table 3) and the corresponding response surface plot (Figure 7) revealed that the lipid and surfactants significantly influenced the drug dissolution rate (P<0.0001).

**Fig. 8.**
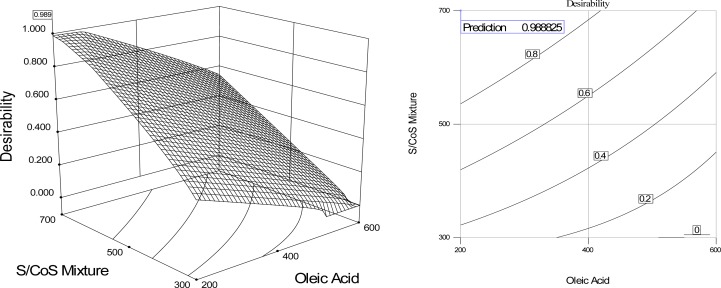
3D surface response plot and contour plot of desirability factor considering maximum ATV release, minimum microemulsion size, maximum transmittance, and minimum self-emulsification time.

Figure 8 shows the 3D response surface plot and contour plot of desirability considering conditions favorable for maximum ATV release. From the graph, it can easily be seen that, in order to achieve maximum ATV release, minimum droplet size along with minimum emulsification time is desired. And to achieve these, OA should be kept in its lower values, whereas the S/CoS should be kept in its highest values (Table 4).

### Stability screening

Optimum levels of oil, surfactant, and cosurfactants are necessary to produce a thermo– dynamically stable microemulsion system. Besides, SEDDS formulations are generally put into hard gelatin capsules as the final dosage form. These two factors play the pivotal role in maintaining the ultimate stability of the final SEDDS dosage form. Liquid-filled hard gelatin capsules are susceptible to leakage, and the entire system has a very limited shelf-life, owing to its liquid characteristics and the possibility of precipitation of the drug from the system.

Due to these reasons, the developed formulations were subjected to accelerated stability testing (40°C/75% relative humidity) to evaluate their stability and the integrity of the final dosage form as well. Physical characteristics (color, visual clarity, phase separation/precipitation) were re-evaluated after one month and three months of formulation-time. After the first month, four out of nine formulations became cloudy which were considered as unstable, where two of them showed precipitation (Table 5). After the third month, three of the four cloudy preparations showed precipitation. So, the rest of the five stable formulations could be the choice for selection, among which SES6 would be the best to be considered for further preclinical study.

## Conclusion

Finally, it can be concluded that dissolution of ATV can successfully be enhanced by incorporating it in SEDDS. Oleic acid, Tween 80, and PEG 400 can be the choices of oil, surfactant, and co-surfactant, respectively, whereas a maximum of 12.5% of the drug can be incorporated safely into the formulation. Pseudo-ternary phase diagram construction has been proven a vital step to generate the optimum ratio of oil/surfactant/co-surfactant. A 3^2^ factorial design without replication has also been proven sufficient to correlate the dependant and independent variables. So, the potential of these formulations for bioavailability enhancement and possible gastric irritation due to the use of large amount of surfactants, needs to be further evaluated by *in vivo* studies.

## Figures and Tables

**Tab. 1.** 3^2^ factorial designs of ATV-SEDDS for 100 mg of ATV: Independent (X) and Dependent variables (Y)

**Batch (SES)**	**Independent Variables**		**Dependent Variables**			

	**X1**	**X2**	**Y1**	**Y2**	**Y3**	**Y4**

	**OA mg**	**Surfactant Mixture (Tween 80–PEG 400) mg**	**ATV release (%) after 10 min mg**	**Microemulsion Droplet Size μm**	**% Transmittance**	**Emulsification Time min**
1	400	300	66.54	20.53	94.95	2.17
2	400	500	81.12	11.345	98.76	1.82
3	400	700	95.54	13.41	97.74	1.41
4	200	300	71.54	15.504	97.32	2.16
5	200	500	89.54	8.811	98.91	1.8
6	200	700	98.78	8.432	99.19	1.32
7	600	300	61.00	27.69	93.50	2.23
8	600	500	72.12	26.941	93.49	1.9
9	600	700	89.54	17.32	95.31	1.45

SES = self-emulsifying system.

**Tab. 2.** Solubility of atorvastatin in different excipients

**Excipient**	**Solubility (mg/mL)**	**Excipient**	**Solubility (mg/mL)**
Arachis Oil	8.8 ± 3.42	Capmul PG8	18.32 ± 4.44
Soybean Oil	8.9 ± 5.13	Cremophor RH 40	19.43 ± 6.17
Castor Oil	9.2 ± 3.10	Cremophor RH 60	21.34 ± 3.85
Oleic Acid	49.23 ± 2.93	Cremophor EL	15.25 ± 4.02
Cremophor CO 40	11.14 ± 5.41	Glycerol	5.91 ± 5.11
Cremophor CO 60	13.54 ± 2.91	Tween 20	32.85 ± 6.69
Water (pH 2.1)	0.02 ± 1.61	Tween 80	38.32 ± 3.41
Water (pH 6.0)	1.21 ± 3.04	PEG 400	40.11 ± 5.7

mean ± SD

**Tab. 3.** Summary of Regression Results for the measured Responses

**Parameters**	**β_0_**	**β_1_**	**β_2_**	**r^2^**	**P**
ATV Release (%) after 10 min (min)	+80.64	−6.2	+14.13	0.98	<0.0001
Microemulsion Droplet Size (μm)	+16.66	+6.53	−4.03	0.85	0.002
% Transmittance	+96.57	−2.19	+1.08	0.74	0.0037
Emulsification Time (min)	+1.81	+0.05	−0.4	0.99	<0.0001

**Tab. 4.** Desirability factors considering the optimum conditions for maximum solubility

**Nr.**	**Oleic acid (mg)**	**S/CoS Mixture (mg)**	**ATV Release after 10 min**	**Microemulsion droplet size (μm)**

1	200	700	100.9	6.0
2	217.7	699.9	100.4	6.6
3	238.4	699.9	99.7	7.3

**Nr.**	**Transmittance (%)**	**Emulsification Time (min)**	**Desirability**	

1	100	1.36	0.989	
2	100	1.36	0.988	
3	99	1.36	0.986	

**Tab. 5.** Results of the stability study of the optimized SEDDS batches

**Batch (SES)**	**After preparation**			**After 1 month**			**After 3 months**		

	**Color**	**Clarity**	**Precipitation**	**Color**	**Clarity**	**Precipitation**	**Color**	**Clarity**	**Precipitation**

1	Light Yellow	Clear	No	Light Yellow	Cloudy	Yes	Light Yellow	Cloudy	Yes
2	Yellow	Clear	No	Yellow	Clear	No	Yellow	Clear	No
3	Yellow	Clear	No	Yellow	Clear	No	Yellow	Clear	No
4	Yellow	Clear	No	Yellow	Cloudy	Yes	Yellow	Cloudy	Yes
5	Yellow	Clear	No	Yellow	Clear	No	Yellow	Clear	No
6	Yellow	Clear	No	Yellow	Clear	No	Yellow	Clear	No
7	Light Yellow	Clear	No	Light Yellow	Cloudy	No	Light Yellow	Cloudy	Yes
8	Light Yellow	Clear	No	Light Yellow	Cloudy	No	Light Yellow	Cloudy	No
9	Light Yellow	Clear	No	Light Yellow	Clear	No	Light Yellow	Clear	No

SES = self-emulsifying system
